# Supplementary material for: Wearable-Based Stair Climb Power Estimation and Activity Classification
Source: Sensors (Basel). 2022 Sep 1;22(17):6600. doi: 10.3390/s22176600 (PMC9459813; doi:10.3390/s22176600)
Supplement: Supplementary file 1 [file sensors-22-06600-s001.zip › sensors-1841658-supplementary.pdf]

Supplementary Materials:

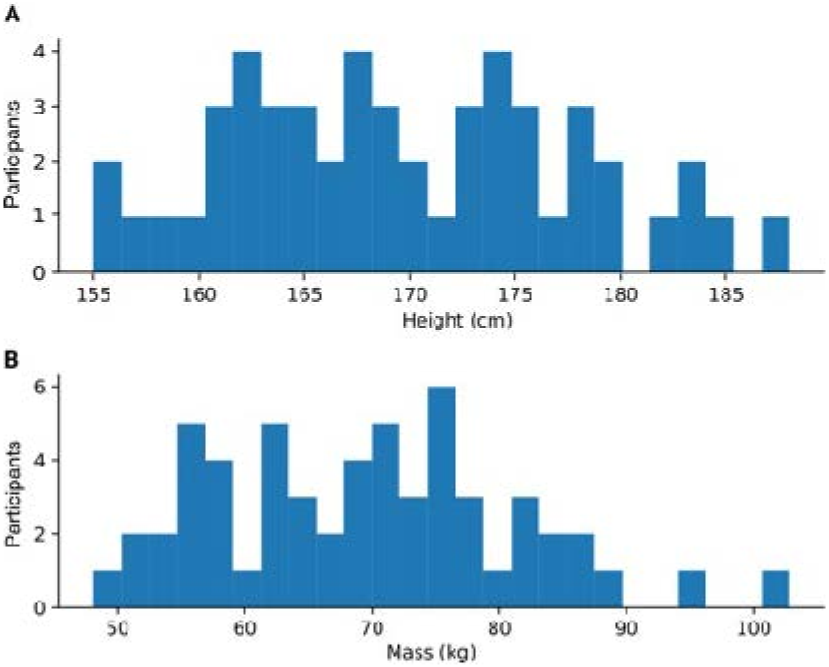

***Figure S1**: **(A)** Distribution of participant height (cm) and **(B)** the distribution of participant mass (kg)*

***Link S1**: (Code Availability) - The StairPy module outlined in this publication is available on GitHub: https://github.com/PfizerRD/Stair_Climbing_DMTI/tree/main/stairpy/StairPy_Notebook.*

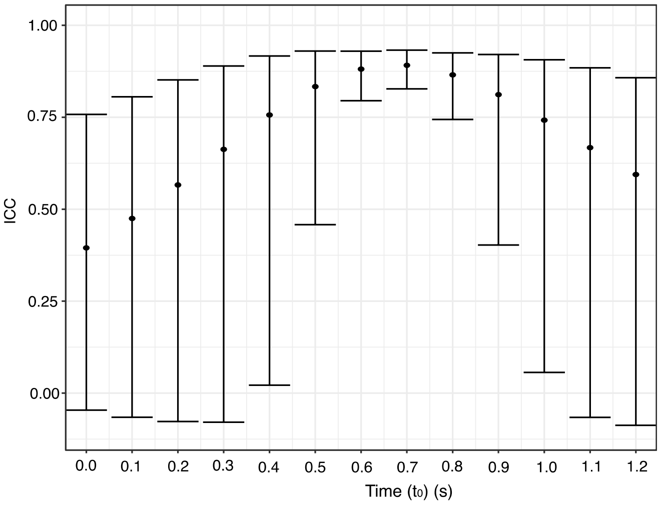

***Figure S2**: Time (sec) to first step (t_0) vs ICC value (Equation 2). This figure shows the relationship between values for the time to first step (t_0) vs the ICC value, which is a measure of agreement between Clinical SCP and StairPy SCP. The time to first step (t_0) shown were applied to all subject's StairPy SCP estimation.*

***Table S1**: Features used for ML classifier training*

Feature	Axis	Sensors	Domain
Median	x, y, z	Accel, Gyro	Time
Mean	x, y, z	Accel, Gyro	Time
Max	x, y, z	Accel, Gyro	Time
Min	x, y, z	Accel, Gyro	Time
Standard Dev	x, y, z	Accel, Gyro	Time
Range	x, y, z	Accel, Gyro	Time
Corr Coef	xy, xz, yz	Accel, Gyro	Time
RMS	x, y, z	Accel, Gyro	Time
Signal Entropy	x, y, z	Accel, Gyro	Frequency
Dom. Freq. Value	x, y, z	Accel, Gyro	Frequency
Dom. Freq. Ratio	x, y, z	Accel, Gyro	Frequency
Dom. Freq. Magnitude	x, y, z	Accel, Gyro	Frequency
Spectral Flatness	x, y, z	Accel, Gyro	Frequency
Variance	x, y, z	Accel, Gyro	Time
FFT Energy	x, y, z	Accel, Gyro	Frequency
Vert Power	y	Accel	Frequency
VAP ratio	z	Accel	Frequency
VMLAP Ratio	x, y, z	Accel	Frequency
Corr power	y, z	Accel	Frequency
Skewness	x, y, z	Accel, Gyro	Time
Kurtosis	x, y, z	Accel, Gyro	Time
Autocorrelation	x, y, z	Accel, Gyro	Time
Interquartile Range	x, y, z	Accel, Gyro	Time
Zero Crossings	x, y, z	Accel, Gyro	Time
20th Percentile	x, y, z	Accel, Gyro	Time
50th Percentile	x, y, z	Accel, Gyro	Time
80th Percentile	x, y, z	Accel, Gyro	Time
